# Between contraception and hormones: a qualitative analysis of the lived experiences of former contraceptive pill users

**DOI:** 10.1080/26410397.2025.2563393

**Published:** 2025-09-22

**Authors:** Jana Niemann, Lisa Glaum, Lea Hofmann, Nadja Freymüller, Liane Schenk

**Affiliations:** a Research Associate, Institute of Medical Sociology (IMS), Interdisciplinary Center for Health Sciences, Medical School of the Martin Luther University Halle-Wittenberg, Halle, Germany. *Correspondence*: jana.niemann@charite.de, jana.niemann@medizin.uni-halle.de; b Student Assistant, Institute of Medical Sociology (IMS), Interdisciplinary Center for Health Sciences, Medical School of the Martin Luther University Halle-Wittenberg, Halle, Germany; c Doctoral Student, Institute for Medical Epidemiology, Biometrics, and Informatics (IMEBI), Interdisciplinary Center for Health Sciences, Medical School of the Martin Luther University Halle-Wittenberg, Halle, Germany; d Research Associate, Institute for Rehabilitation Medicine, Interdisciplinary Center for Health Sciences, Medical School of the Martin Luther University Halle-Wittenberg, Halle, Germany; e Doctoral Student, Department of Sustainable Environmental Health Sciences, Medical School OWL, Bielefeld, University, Bielefeld, Germany; f Professor of Health Services Research, Specialising in Vulnerable Groups, Charité – Universitätsmedizin Berlin, Institute for Medical Sociology and Rehabilitation Science, Berlin, Germany

**Keywords:** contraception, cessation, discontinuation, contraceptive choice, reproductive justice

## Abstract

Contraceptive decision-making is an ongoing process that affects reproductive life and involves method uptake, use, and discontinuation. Contraceptive pills have been widely studied for their side effects, lived experiences, and links to biomedicalisation. However, there is a lack of research that integrates the entire subjective contraceptive pill experience from implementation to the period after discontinuation. This study explores the lived experience of pill use in Germany through 19 oral contraceptive pill biographies using thematic analysis, a deductive-inductive, iterative coding approach, and team-based discussions. This work is grounded in the theoretical framework of the contraceptive journey and feminist inquiry. Our analysis illustrated the complexity of pill use, with frequent switching, stopping, and restarting influenced by biopsychological issues, normalisation of use, and adverse effects. Attitudes towards hormones and pregnancy risk evolve over time and age. Former users consider their contraceptive journey to be successful in preventing pregnancy, but desire more male contraceptive options and improved information and counselling services. These findings emphasise the importance of prioritising former users’ informal knowledge and lived experiences in future research, policy, and practice. This approach can support healthcare providers in incorporating individual and diverse health needs, aligning with the principles of reproductive justice.

## Plain language summary

This study examines the experiences of former contraceptive pill users in Germany. It focuses on their entire journey with the pill, from when they first started taking it to after they stopped. The researchers interviewed 19 former pill users and analysed their stories. Key findings include: 1. Pill use is complex, with women often switching, stopping, and restarting due to various factors. 2. Women’s attitudes towards hormones and pregnancy risk change over time. 3. Former users generally feel successful in preventing pregnancy but want more contraceptive options for men. 4. There’s a need for better information and counselling about contraceptive pills. The study emphasises the importance of considering women’s personal experiences and informal knowledge in future research, policy-making, and healthcare practices. This approach can help healthcare providers better support individual contraceptive needs and promote reproductive justice.

## Résumé

La prise de décision en matière de contraception est un processus continu qui affecte la vie reproductive et comporte l’adoption, l’utilisation et l’arrêt d’une méthode. Les pilules contraceptives ont été largement étudiées pour leurs effets secondaires, leurs expériences vécues et leurs liens avec la biomédicalisation. Néanmoins, rares sont les recherches qui intègrent la totalité de l’expérience subjective avec la pilule contraceptive depuis sa mise en place jusqu’à la période suivant son arrêt. Cette étude explore le vécu de l’utilisation de la pilule en Allemagne avec 19 biographies de pilules contraceptives orales, en utilisant une analyse thématique, une approche de codage itératif déductive-inductive et des discussions par équipe. Ce travail s’appuie sur le cadre théorique du parcours contraceptif et la recherche féministe. Notre analyse a illustré la complexité de l’utilisation de la pilule, avec de fréquents changements, arrêts et reprises influencés par des problèmes biopsychologiques, la normalisation de l’utilisation et les effets secondaires. Les attitudes envers les hormones et le risque de grossesse évoluent avec le temps et l’âge. Les anciennes utilisatrices considèrent que leur parcours contraceptif a été efficace pour prévenir les grossesses, mais souhaitent davantage d’options contraceptives masculines et de meilleurs services d’information et de conseil. Ces résultats soulignent combien il est important de donner la priorité aux connaissances informelles et aux expériences vécues des anciennes utilisatrices dans les recherches, politiques et pratiques futures. Cette approche peut aider les prestataires de soins à intégrer les besoins de santé individuels et divers, en les alignant sur les principes de la justice reproductive.

## Resumen

La toma de decisiones sobre la anticoncepción es un proceso continuo que afecta la vida reproductiva y abarca la aceptación, el uso y el abandono del método. Las píldoras anticonceptivas han sido estudiadas extensamente por sus efectos secundarios, vivencias relacionadas con ellas y vínculos con la biomedicalización. Sin embargo, se carece de investigaciones que integren toda la experiencia subjetiva con las píldoras anticonceptivas desde su inicio hasta el período después de su descontinuación. Este estudio explora las vivencias relacionadas con el uso de la píldora en Alemania por medio de 19 biografías sobre píldoras anticonceptivas orales, utilizando el análisis temático, un enfoque de codificación iterativa deductiva-inductiva y debates en equipo. Este trabajo está basado en el marco teórico de la trayectoria anticonceptiva y la investigación feminista. Nuestro análisis mostró la complejidad del uso de la píldora, en que el cambio de método, cese y reinicio del uso se ven afectados por asuntos biopsicológicos, la normalización del uso y efectos adversos. Las actitudes hacia las hormonas y el riesgo de embarazo evolucionan con el tiempo y la edad. Las antiguas usuarias consideran su trayectoria anticonceptiva como un éxito en la prevención del embarazo, pero desean más opciones anticonceptivas masculinas, así como información y servicios de asesoramiento mejorados. Estos hallazgos hacen hincapié en la importancia de priorizar el conocimiento informal y las vivencias de las antiguas usuarias en futuras investigaciones, políticas y prácticas. Este enfoque puede apoyar a profesionales de salud en incorporar las necesidades de salud individuales y diversas y alinearse con los principios de la justicia reproductiva.

*“I feel like I know something again: Who I am. And no longer: What could be a side effect of the hormonal pill? Instead, I know that when I feel down, I truly am down.”* (Lola*)

## Background

Lola’s reflection on the impact of discontinuing the contraceptive pill on her life and health highlights how she feels more authentic and has gained insight about the pill’s effects. Through an analysis of Lola’s and other contraceptive pill biographies in Germany, this article explores how the significance of a contraceptive method is influenced by lived experiences and evolving values and norms surrounding the pill.

Birth control is a fundamental sexual and reproductive right^1^ that enables individuals globally to make decisions about their reproductive lives and sexual health. The oral contraceptive pill (OCP), has historically empowered women† by transferring contraceptive control from men to women, thus enhancing women’s choices in education, career, and family planning.^2^ This shift has also altered social gender norms by placing contraceptive responsibility on women.^3,4^

Within this spectrum of contraceptive choices and responsibilities, research has shown that contraceptive choices are complex and nonlinear.^5–7^ Downey et al. argue that contraceptive decision-making is not a straightforward process that ends with the most effective method, but rather an iterative, relational, and reflective journey. Instead, women revisit their choices, with knowledge accumulated along the way.^5^ Based on this, Simmons et al. expanded this work and termed this process the contraceptive journey, which is a dynamic, cumulative process throughout reproductive life.^7^ Their work underscores the importance of method neutrality, the normalcy of changing methods over time, and the impact of past experiences (path dependence) on future decisions. This user-centred framework challenges traditional, one-size-fits-all approaches by emphasising the complexity and evolving nature of contraceptive use. Despite this variability, D’Souza et al.’s review highlights that factors influencing contraceptive use are consistent across diverse cultural and socioeconomic settings globally.^8^ Among these factors, the OCP’s side effects^9^ and lived experiences^3,10^ significantly influenced this journey. Users may experience various positive ^11, 12^ and negative side effects^13–15^ depending on the type of OCP.

However, quantitative studies struggle to measure embodied and lived experiences.^10,16^ This may partly explain the persistence of conflicting epidemiological research^17^ and poor-quality evidence^18^ regarding the side effects. Qualitative scholars have highlighted how negative side effects impact pill users’ lives.^3,10,19–21^ Research indicates a disconnect between healthcare providers and patients in contraceptive counselling,^22–24^ which, along with changing life circumstances, may lead to the discontinuation of the OCP.

The primary reasons for discontinuation include adverse side effects^9,16,25–28^ and changes in pregnancy risks.^9,16,25,26^ However, qualitative research results emphasise that the discontinuation process is more complex. A focus group study on OCP discontinuation suggested that the growing awareness of side effects throughout implementation may influence this decision significantly.^27^ Additionally, communication with peers and healthcare providers alongside online information plays a crucial role.^27^ Applying a reproductive justice framework, a systematic review identified the desire for natural, hormone-free contraception and the perceived invalidation of side effects by health professionals as key factors in rejecting hormonal contraception.^9^ Social media influencers share discontinuing hormonal birth control for *“being more natural”* and due to negative side effects.^29,30^ These findings highlight the complex nature of the decision to stop using OCPs, driven by side effects, personal health values, and social and medical advice.

The scant research on post-discontinuation globally has concentrated on menstrual regularity, return, and fertility restoration.^31,32^ Few studies have examined the post-discontinuation effects beyond fertility restoration, which is especially critical from a feminist viewpoint, as it limits the value of the bodily experiences to the body’s reproductive capability. Analyses of self-uploaded YouTube videos by social media influencers have explored their experiences with OCP (dis)continuation.^29,30^ A study of German YouTube videos revealed that influencers reported facial skin impurities (108/158), reduced mood swings (47/158), hair loss (42/158), and weight loss (36/158) after discontinuation.^30^ However, social media analyses may be biased because of the non-representative content. Otte et al. noted that women in their qualitative study experienced multiple side effects simultaneously, with irregular bleeding being common, and were uncertain if biopsychological issues were related to discontinuation.^27^

Research indicates that (dis)continuation of the OCP is a complex, context-dependent cumulative process influenced by individual experiences^5,9,10^ and the power of society and (health) institutions.^3,33–35^ Numerous epidemiological studies have examined potential side effects, and some qualitative studies have explored personal experiences with the pill. However, comprehensive perspectives on OCP biographies from initiation to post-discontinuation are scarce.^10,27^ From a feminist perspective, this sidelines (former) pill users’ broader health and autonomy, reinforcing traditional views that prioritise their reproductive roles. Therefore, this article aims to explore former pill users’ perceptions of and experiences with the OCP from initiation to post-discontinuation in Germany, addressing the following research questions: (1) What are their understandings, attitudes, beliefs, and perceptions of the OCP throughout its use? (2) How do they describe their experiences with the (dis)continuation of the OCP?

### Theoretical and conceptual framework

Our theoretical framework is derived from the contraceptive journey (CJ) theory^6^ and current research on OCP (dis)continuation. The CJ framework provides a valuable lens through which to examine the complex process of OCP (dis)continuation. Simmons et al. observed that contraceptive decision-making is an ongoing, multifactorial process that influences reproductive life.^6^ Their qualitative study developed the CJ, which describes the temporal nature of contraceptive decision-making and is divided into four phases: (1) identification of needs, (2) method initiation, (3) method continuation, and (4) method cessation. These phases are shaped by five decisional influences: (a) physiological factors, (b) values, (c) experiences, (d) circumstances, and (e) relationships.

Building on this foundation, our adaptation of the CJ framework introduces two key modifications. First, based on a literature review, we added a fifth phase, *“post-cessation,”* to the framework to account for decisions, reflections, and possible transitions following discontinuation. Furthermore, dimension (a) will be revised from *“physiological factors”* to *“biopsychological factors,”* explicitly integrating both physiological and psychological experiences.

**Figure 1.**
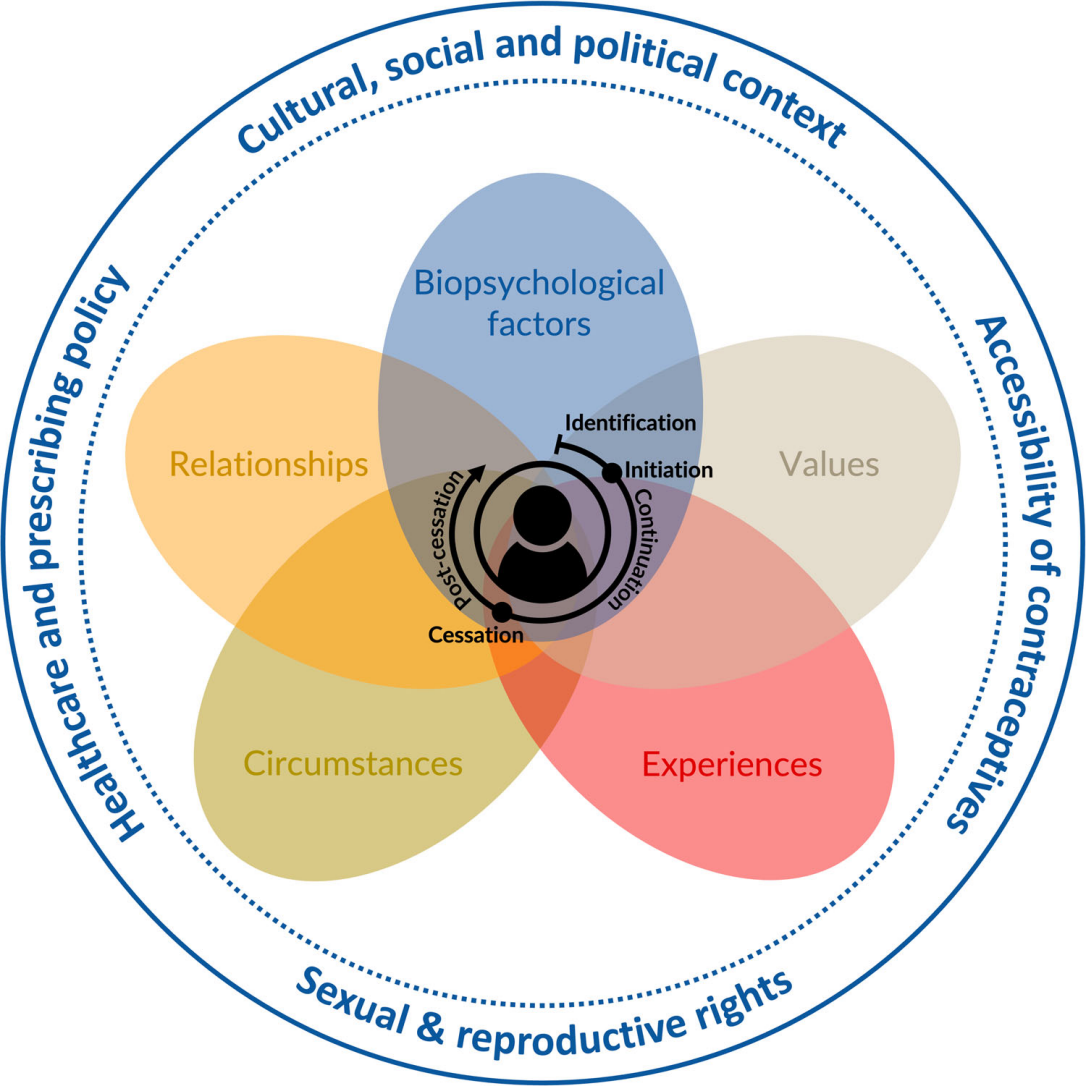
Conceptual framework of the contraceptive journey with the pill (Own Illustration) Note: Figure 1 illustrates the theory of the contraceptive journey^6^ under theoretical considerations of the literature on oral contraceptive pill (OCP) (dis)continuation. The OCP life cycle consists of five phases: (1) identification of OCP, (2) initiation of OCP, (3) continuation of OCP, (4) cessation of OCP, and (5) post-cessation of OCP. These phases are shaped by five influential factors: (a) biopsychological factors, (b) values, (c) experience, (d) circumstances, and (e) relationships. The surrounding structural and societal factors may affect individual experiences.

Figure 1 illustrates the adapted conceptual model as a dynamic cyclical process influenced by individual and contextual factors. This is vital for analysing contraceptive biographies, highlighting the personalised period from initiation to discontinuation. The centre of the graphic depicts the period from initiation to discontinuation, highlighting the personal and historical nature of these time dimensions. These decisions are shaped by five intersecting domains: biopsychological factors, experiences, values, circumstances, and relationships. These domains interact with each phase of the journey, shaping and reshaping decisions over time. The outer circle illustrates the structural and power dimensions crucial to the context of the CJ with the OCP.

Overall, this adapted framework allows for a nuanced and user-centred analysis of contraceptive biographies. It emphasises the complexity, fluidity, and path-dependent nature of contraceptive use over the reproductive life course, challenging the linear and static understanding of (dis)continuation.

## Methodology

Our theoretical framework incorporated a feminist critical lens, which prioritises women’s experiences and voices in research,^36^ seeks to eliminate gender disparities, and challenges traditional androcentric perspectives.^37^ Using this qualitative feminist perspective, we aimed to highlight the lived experiences of individuals managing gendered contraceptive responsibilities, specifically those who (dis)continued the OCP.

### Setting

In Germany, OCPs are typically prescribed by gynaecologists but can also be prescribed by general practitioners, with medical counselling guidelines which have been established since 2020.^38^ Prescriptions for OCPs have consistently declined over the past twenty years, accompanied by a rise in negative attitudes towards hormonal contraceptives.^39^

### Recruitment, data collection, and participants

This study received approval from the Ethical Review Board of the Medical Faculty at the Martin Luther University Halle-Wittenberg (Reg-Nr. 2021-034, date 07/04/21). The protocol was published prior to the interviews.^40^ We followed the Standards for Reporting Qualitative Research^41^ to report our study findings (cf. Supplementary File 1). The primary researcher (JN) conducted 19 episodic interviews with former OCP users from the 15th of December 2022 to the 13th of September 2023. The study included German-speaking individuals aged 19–38, consisting of cisgender women and one non-binary person, all residing in Germany at the time of the interviews, except for one participant who had relocated abroad but continued consulting a German gynaecologist. Another participant, originally from Italy, had moved to Germany and transitioned to German gynaecologists, being proficient in German. Participants’ educational levels ranged from secondary school certificates to master’s degrees, with most reporting good to very good health. One participant had children, and another was pregnant at the time of the interview, with at least six months between discontinuation of OCP and pregnancy. The median time since their last discontinuation was six months. More than half had initiated the OCP at least twice, resulting in a cumulative 189.25 years of OCP experience. Detailed participant characteristics are available in Table 1.

**Table 1.** Characteristics of the interview sample consisting of former pill users, *N* = 19

Demographic variable	Former pill users
**Gender**	
Cisgender female	18
Non-binary	1
**Sexual orientation**	
Heterosexual	17
Pansexual	1
Bisexual	1
Non-labelled	1
**Age (median)**	27
<25	3
25-34	13
>35	3
**Birth country**	
Germany	18
Italy	1
**Ethnicity**	
White	19
**Educational level**	
Secondary school certificate^a^	5
Subject-related entrance qualification^b^	1
Higher education entrance qualification (A-levels)^c^	4
Bachelor studies	6
Master studies	3
**General health condition**	
Very good	5
Good	11
Average	3
Poor	0
Very poor	0
**General implementation information**	
Time since discontinuation (months, median)	6
Total years of experiences	189,25
**1st implementation**	
Age at 1st initiation (median)	16 (13; 22)
Age at 1st discontinuation (median)	23 (18; 36)
Length of 1st implementation (years, median)	6 (2; 20)
**2nd implementation (n=11)**	
Age at 2nd initiation (median)	25 (23; 37)
Age at 2nd discontinuation (median)	27 (26; 38)
Length of 2nd implementation (years, median)	2 (1; 5)
**3rd implementation (n=3)**	
Age at 3rd initiation (median)	34 (25; 37)
Age at 3rd discontinuation (median)	35 (30; 37)
Length of 3rd implementation (years, median)	1 (0,25; 5)

^a^ “*Realschule*“; ^b^ “*Fachhochschule*“; ^c^ “*Abitur*“

JN recruited participants through social media forums and snowball sampling within personal social networks. Participants self-selected and emailed JN. After confirming eligibility, JN sent detailed study information, a letter of informed consent, and a data protection statement. Upon agreeing to participate, JN offered a brief telephone call to address queries and schedule interviews. All interviews were conducted online. Due to the online format, all participants provided verbal informed consent prior to the episodic interview. Episodic interviews collect detailed accounts of specific life events, including experiences, emotions, and perceptions. JN created a semi-structured interview guide focusing on the experiences and decision-making processes related to the (dis)continuation of the OCP. This guide was discussed with the research team and qualitative method groups at two German universities. Questions were drafted based on a previous study of German YouTubers’ (dis)continuation experiences^30^ and existing literature. The guide included questions about participants’ connection to the pill, initiation of the OCP, counselling with gynaecologists, implementation experiences, discontinuation process, post-discontinuation experiences, and changing attitudes towards hormonal contraception. JN reviewed relevant questions for each implementation and discontinuation if a participant had had multiple experiences. JN conducted two pilot interviews before the data collection. The research team iteratively modified the interview guide, using a demographic questionnaire post-interview to examine participants’ characteristics. Data collection ceased upon achieving thematic saturation, with a minimum target of ten interviews. Saturation was considered reached if, after three additional interviews, only rare new phenomena or cases emerged, such as shared weaning experiences. The stopping criterion was evaluated after each subsequent interview (i.e. 11, 12, 13, etc.),^42^ and assessed by reviewing transcripts during data collection.

### Analysis

The interviews lasted an average of 49 minutes and were audio-recorded and transcribed verbatim by a professional. Transcripts were analysed using MAXQDA with a deductive-inductive thematic analysis approach. Initially, we read all the transcripts and wrote a summary for each person, including key aspects and characteristic quotes. JN and LG developed a codebook with main deductive codes based on the CJ theory and the interview guide. During the coding process, they summarised the themes reconstructed from the transcripts in inductive codes. Both researchers coded all transcripts separately. They met for weekly discussions to refine their inductive codes, resolve discrepancies in coding, and practice reflexivity in their data analysis process. Following each session, an updated codebook was used to code the next interview. After finalising the coding process, JN identified common patterns and subthemes within the code set and between subgroups of interviews (e.g. the number of initiations/discontinuations). She selected illustrative quotes for each subtheme. Both JN and LG reviewed all the themes, and JN created a thematic map (cf. Supplementary Figure 1).

### Positionality and reflexivity

The research team comprised white female German researchers. The principal academic researcher (JN) holds a master’s degree in European Public Health, has personal experience with OCP (dis)continuation, and is skilled in qualitative research. While this study adopts a feminist critical lens, it does not include formal public and patient involvement. When the first author began her PhD, the study was not originally designed with a feminist methodological framework, and limited resources further constrained the opportunities for public and patient involvement. However, her personal experience with OCP discontinuation and a strong belief in reproductive health as a feminist issue shaped the research from the beginning. Over time, her approach evolved, and she chose to analyse the data through a feminist lens to better centre participants’ voices and lived experiences.

A master-level student assistant (LG) has (dis)continued the OCP and has qualitative research experience. A medical student assistant (LH), who has worked on other qualitative projects, is a former OCP user. The third academic researcher (NF) is also a former OCP user and has experience in qualitative research. The second academic researcher (LS) is a professor with over 15 years of postgraduate qualitative research experience.

## Results

Proceeding with our conceptual framework, we found a chronological rhythm throughout the OCP biographies, shaped by biopsychological factors, values, relationships, circumstances, and experiences. These factors often overlap, as shown in Figure 1, highlighting the dynamic nature of the pill journey.

### Initiation process

*“My menstruation was the only reason”* – Biopsychological factors

Physiological factors, such as menstrual pain, irregular periods, and skin impurities, influenced the initiation process significantly. Heather, for example, experienced severe pain and cramps, stating she *“could hardly stand up,”* while Natalia faced *“skin problems, especially on the back and décolleté.”* Regarding subsequent uses of the OCP, participants primarily cited reasons such as cysts, polycystic ovary syndrome, and adverse reactions to other contraceptive methods.

*“For peace of mind”* – Values

Interviewees felt social pressure to take the OCP during the initiation phase, often making decisions without much thought. Lola notes: *“It was common sense for women to take the pill. Everyone did this at the school. Many, like a classmate of mine, took it because they didn’t get their period and were just prescribed the pill.”* This perception renders the pill a common and unquestionable contraceptive. Lily recalls, *“My mom was on the pill and didn’t think much about it. It was an easy and safe option. Almost everyone in my circle of friends was on it.”* Many took the OCP naturally to securely prevent pregnancy: *“For me, it was clear: You do not get pregnant at school, period! Hence, you take the pill, period!”* (Olive).

Olive’s description underscores the significance of pregnancy prevention during that life stage, with the pill representing the freedom to engage in sexual activities.

*“The easier method”* – Experiences

Experiences with other contraceptives, primarily condoms, influenced the initial adoption of the pill. Luna, for example, expressed dissatisfaction with condoms due to their tendency to *“slip and rupture.”* Some interviewees cited convenience as a reason for choosing the pill over other methods.

Regarding subsequent initiations, participants were already familiar with the pill’s safety and ease of use. Julie, who faced difficulties using condoms because her partner had erectile dysfunction, eventually chose the pill as the easiest method. She noted, *“I tried to stop in between, but using a condom for contraception is not viable. My partner also suffers from erectile dysfunction. This was difficult to overcome with a condom. So, I resumed hormonal contraception repeatedly.”*

*“The classic sexual education class”* – Circumstances

One of the main factors in identifying OCPs as a birth control method is the educational system. Many people learned about OCPs and other birth control methods in school sex education classes. Rachel recalled that in 2003, when she first used the OCP, she had no information to self-educate: *“I was prescribed the OCP at 13 in 2003, a time when information on the pill and its health implications was limited.”* By contrast, Sophia informed herself via social media: *“I even heard the debate about whether it was good for the woman or not. And yet I decided to do it because I thought it was appropriate for my situation.”* These narratives illustrate the intergenerational diversity in our sample, which may have influenced OCP experiences.

*“The first recommendation was the pill”* – Relationships

Relationships with partners, family, friends, and gynaecologists greatly influenced the decision to start the OCP. Participants cited initial relationships as a reason for choosing the pill, with partners typically not participating actively in the decision.

*“[…] I did it without him knowing, so to speak. […]. There was a big situation afterward before we slept together for the first time, I said to him [laughs] ‘I will take the pill if you want’”*. (Lola)

Lola’s narrative illustrates how her romantic relationship influenced her decision to use the OCP, highlighting the reproductive responsibility our respondents assume within their sexual lives.

Respondents highlighted the importance of the mother’s role, noting discussions with their mothers about contraceptive methods. Lola mentioned her mother’s attitude towards preventing pregnancy: *“My mom did not truly care how I used contraception. The main thing was that she had done her motherly duty and knew I would not become pregnant.”* Conversely, two respondents noted their mothers’ hesitance about their daughters’ use of the OCP. Lydia stated, *“My mom was not like: ‘Definitely take this.’ She was always a bit skeptical. So I was not pushed in any direction. It was more like: ‘Yeah, I’d like that too.’”*

Gynaecologists were crucial in identifying and prescribing the OCP. Most interviewees reported that, during their first visit, they were promptly prescribed the pill for physiological issues and/or contraception. Participants typically adhered to the gynaecologist’s advice without considering alternative contraceptive methods.

*“[…] So I discussed it with my gynaecologist. And the first recommendation was really the pill. I don’t think I was really told about any other method when I went to the doctor. To be honest, I didn’t ask about any others. So for me, it was kind of in my head that you just take the pill when it becomes relevant, right? And that’s why I got it right away.”* (Emma)

Emma’s description reveals the influence of gynaecologists on contraceptive choices and the users’ blind trust in them (their gynaecologists).

### The implementation

*“Between the positive and negative”* – Biopsychological factors

The pill’s use was linked to both positive and negative effects that impacted the individual’s experience significantly. Positive effects included relief from menstrual issues and improved skin, as Natalia noted: *“It was just something completely different for me to lie on the beach or go swimming. I would not have dared before. And that was the moment when I thought, It was the best decision of my life to take the pill.”* However, nearly all former pill users reported problems, such as mood swings, depressive symptoms, reduced libido, migraines, headaches, weight gain, and bleeding. Mood swings, for example, were described as *“freaked out over nothing”* (Luna), *“I just got frustrated very quickly”* (Olive), and *“became very emotional”* (Sophia). Ida described her experience with the *“lactation pill”* (German *“Stillpille”*) as: “*Yeah, I was bleeding, and I felt like I had my period all the time […]. And it was all just a kind of silly.”* These issues varied, reflecting the diversity of OCPs taken by our participants.

Continuation issues occasionally prompted switching between different OCPs. Participants mentioned trying alternative OCPs recommended by their gynaecologists, hoping *“[…] that might be a better fit”* (Lydia). Some noted that switching pills resulted in fewer problems.

*“Feeling like an adult”* – Values

Former pill users perceived taking the pill as a sign of maturity and responsibility, viewing it as a step towards adulthood and valuing its reliable contraceptive protection. Sophia explains: *“Yeah, I’m growing up now. I have to take care of this and make these decisions. Consequently, it was almost a step toward growing up [laughs].I”* Adverse effects and questioning the impact of OCP were initially considered unimportant.

**Jana:** *And did you think about the consequences of taking the pill?*

**Natalia:** *Not at all. I thought: ‘Everybody does it. The doctor said I should take it. What could go wrong? [laughs]*. (Natalia)

*“This was always very present”* – Experiences

The former users described their experiences with the pill and the impact it had on their daily lives. Some considered it stressful and inconvenient because of its daily requirements and the occasional difficulty in obtaining it. Concerns arose when they were ill or forgot to accept it. Overall, the regular use of the pill influenced their daily routines significantly. Fannie highlights both its advantages and drawbacks:

*“I mean, sexual relationships were certainly more spontaneous. That was kind of the good side. […] And then the bad side was that whenever I slept somewhere or something, I truly always thought: ‘Did I take my pill? Did I take the pill on time?’ That was always very present […]”* (Fannie)

This quote highlights the conflict Fannie and other interviewees experienced with the OCP. She describes the pill’s safety, which we discussed in the values section, and how it made her feel freer during sex. However, she also felt insecure about the pill regimen. Conversely, other respondents mentioned positive aspects, such as postponing periods and taking the pill became mostly automatic, as Emma described: *“Yes, I definitely got more confident. At some point, I realized: ‘Okay, it is going well. I do not have to worry about it at all’”.*

*“At some point we just wanted to do it without condoms”* – Relationships

Most respondents were in some form of relationship, committed or not, while using the pill. The pill provided security for their sexual relationships. In noncommitted relationships, participants sought additional pregnancy prevention alongside condoms. In committed relationships, they preferred sex without condoms. Some also ceased contraception at the end of a relationship and resumed it at the start of a new one. Lydia stated, for instance: *“After that [the relationship], I stopped. And when I met my new boyfriend, three years later, and when I was sure it was going to be something long-term, I decided to do it again.”* Lydia emphasised the pill’s contraceptive benefits, with her relationship status influencing its use. This highlights the complexity of contraceptive histories.

Some respondents reported that their gynaecologists dismissed their side effects or concerns about the pill, insisting that they continued using the OCP. Lily described her gynaecologist’s responses as follows:

“*And then I had a gynaecologist who was totally enthusiastic about the pill, who was horrified that everyone was now starting to get off the pill […]. So she did not respond to me at all.*” (Lily)

### The discontinuation process

*“*It was enough for me*”* – Biopsychological factors

Biopsychological factors accumulated over time, affecting the quality of life. The side effects of medication eventually became intolerable: *“I want to get rid of these symptoms and I no longer take it [the pill]”* (Natalia). This resistance to systematic prescription was echoed by Julie: *“And then I said to myself: ‘Now, this far and no further. That is enough!’”*

Several former users expressed a desire to understand their bodies better and determine whether the negative side effects were linked to the OCP: *“And I kind of wanted to know, after so many years, okay, what are the real side effects?”* (Lola). This also reflects the authenticity that she felt during the implementation process.

Some respondents experienced fear and uncertainty before discontinuing treatment because of their potential physical symptoms. Coral, for instance, diagnosed with PCO, feared the symptoms would worsen without the pill: *“I was afraid to stop taking the pill because I was afraid that the symptoms of PCO syndrome would worsen again.”* Similarly, Lily expressed concerns about contraception: *“Initially, there was some uncertainty regarding contraception, including how it works and when necessary. It is important to be aware of the days when contraception is needed and to use it consistently.”* These accounts highlight a critical stage in individuals’ lives where uncertainties about physical development and future contraception play significant roles.

*“Finding out about hormones”* – Values

Interviews revealed that discontinuing the OCP is influenced by evolving attitudes towards hormones and the possibility of pregnancy. Interviewees preferred a hormone-free lifestyle and sought more information on hormones. Ida exemplifies this: *“Because I had already started to find out a bit about hormones and things like that. Maybe it was not necessarily a good idea to keep pumping yourself full of hormones if the actual reason for taking them was not there at the time”.* Stopping reproduction for older respondents marked a transition in reproductive history. Some couples decided against having children and chose vasectomy or sterilisation. Rachel explains, *“[…] We came to the final decision: Okay, do we still want to deal with the issue of children or not? Then, we had longer discussions, and we both concluded, ‘No, children are not an option.’”* Others did not plan an immediate pregnancy but were no longer concerned about unexpected pregnancies. Fannie noted, *“If I want kids one day, I do not want to find out the day I have problems with it [laughs].”* Therefore, the ability to conceive remains a significant factor.

*“Between curiosity and fear”* – Experiences

Some respondents researched the effects of stopping birth control, such as hair loss, weight gain, and acne, using online resources (e.g. YouTube, Facebook), medical advice, and scientific articles.

“*I then looked up a lot of information on the internet about what happens to your hormones in your body when you stop taking the pill; why it can lead to effects such as psychological stress or skin problems or sleep disorders or whatever, why it actually happens.*” (Helena)

Helena’s search for information also revealed curiosity and excitement regarding her discontinuation. Emma shared a similar sentiment: *“I guess I was truly curious: How do I feel without the hormones? I just wanted to try it. It was not fear; it was not stress; it was mostly curiosity.”*

Interviewees also expressed a fear of discontinuation. Coral, for instance, felt anxious because of her pre-pill experience. She lacked a menstrual cycle and worried that *“male”* hormones might increase post-discontinuation, potentially enhancing hair growth: *“Of course, I was also afraid of hair growth. Exactly, these are things. So yeah, I would say I have a queasy feeling, yeah.”*

*“The (in)security in reproductive lives”* – Circumstances

During the implementation of the OCP, respondents transitioned to a more secure life stage in terms of age, employment, financial stability, and relationship status. Lydia, for example, ceased using the OCP as she felt she *“no longer needed it”* after completing her studies and securing employment. The significance of a secure life stage in perceiving oneself as ready for pregnancy reflects the societal discrimination and insecurity faced by young parents. A young pregnancy is closely associated with life risks, and support systems for parents are inadequate.

*“Sharing a decision?”* – Relationships

Respondents felt supported by their partners in deciding to stop using the pill, with open communication about side effects and shared decision-making. Reproductive responsibility was negotiated within relationships, with some partners actively researching the side effects and aiding the decision process. Partners played a crucial role in the provision of reflection and reassurance. Heather stated, *“Yes, he [her partner] always encouraged me and said, ‘No matter what you decide. I always have your back. We will always do it together.’ It was totally collaborative, and we made the decision together.”* Some respondents noted that the decision to withdraw was entirely theirs alone: *“To be honest, it is completely up to me because he is not medically savvy […]. We discuss it actively. He’s not uninterested and very supportive, but even more helpless than I am”* (Olive). Conversely, other participants reported that their partners actively researched the side effects and supported the decision-making process. Luna described, *“My hypochondriac boyfriend brought in many [laughs] medical concerns, and, based on what we thought were good sources, we decided the risks of a medical problem were too great”.* Some former pill users discontinued treatment after their relationships ended, particularly during their first cessation.

Many noted that some friends stopped taking OCPs at around the same time. In their later reproductive years, respondents reported a significant decrease in friends using OCPs. Hannah viewed her cessation as a collective experience with a friend:

*“With one of my friends, she wanted to stop taking the pill because she still had a relatively low libido and said that she definitely wanted to try […] and we both did. That’s how I found out about it through her, and then it was a little bit of an influence.”* (Hannah)

She noted that her friend influenced her decision-making, a sentiment echoed by other respondents who mentioned hearing negative stories about the OCP in their social circles.

During the cessation process, consultation with gynaecologists varied; some respondents sought advice, while others bypassed it because of a perceived lack of support. Some, for example, described that their gynaecologist agreed to discontinue treatment when the side effects were too severe. By contrast, Helena noted, *“In fact, I pretty much bypassed my gynaecologist [laughs] because I just knew she probably would not be much help”.* A neglect of side effects during implementation often leads to mistrust of the gynaecologist during both the discontinuation process and future interactions.

### Post-cessation

*“From the unknown into the normal”* – Biopsychological

Participants reported positive changes after discontinuing OCPs, including improved mood, increased libido, relief, and a sense of freedom. Julie noted, *“The depression has disappeared, which is incredible. I used to think it was a normal part of me, but now I realize that was not the case”.* Similarly, some participants felt more balanced and connected to their bodies, which enhanced their happiness and sense of self. Emma said, *“I immediately felt better and more balanced, which made me happier again”*. Those who experienced a loss of sex drive during OCP use found that it returned after discontinuation, often linked to a sense of relief and enjoyment. Lydia remarked, *“That was also the case with this one: You feel like having sex again. I hadn’t had that feeling for ages before, and I thought: I was back in puberty or my early 20s [laughs]”.* However, two participants experienced negative mood changes. Helena, who discontinued OCPs twice, had severe mood changes after her first discontinuation, leading to reinitiation*: “I cannot take much more of this. I did not know where to go or how to get help quickly, so my only option was to take the pill again since I had it at home”.* She felt overwhelmed and sought to refocus alone. This opportunity came in the form of the OCP.

After discontinuation, the participants observed positive physiological changes, such as reduced headaches. Lily, for example, began ovulating: *“I was quite surprised, I felt myself ovulating for the first time”.* This indicates bodily reconnection after cessation, a sentiment shared by others. The return of menstruation was also significant. Some experienced their first menstruation soon after discontinuation, often feeling relief, and describing it as: *“No problem. It went well”* (Olive) or *“unspectacular”* (Rachel). Others noticed the absence of their period, expressing a desire *“not to stress it”* (Luna), though this absence often suggested bodily dysfunction. Lydia, whose period regularised after six months, felt relieved and *“normal again”.* Regular menstruation was, thus, linked to a normally functioning *“female”* body. Some also reported increased bleeding, cramps, and pain post-cessation, but these were considered *“normal”* rather than negative, as Lola stated, *“So, I […] feel completely normal at all times”.* Some only had intense cramps during their first post-cessation period, after which things *“levelled off relatively normally”* (Julie). Negative changes included oily skin, blemishes, oily hair, and hair loss. Extreme hair loss or skin issues made some feel *“automatically […] a bit more uncomfortable”* (Lily), *“not comfortable”* (Helena), and were *“annoying”* (Coral). Others experienced milder symptoms that were *“nothing that would be a burden”* (Fannie).

*“Between contraceptive method and hormones”* – Values

Former users initially regarded the OCP as a safe contraceptive and means of sexual freedom. However, after stopping their use, they experienced conflicting emotions due to negative experiences and began to perceive it as medication. Olive described: *“Initially, it was contraception, but now it is clear to me that the pill is a drug and nothing else. It is a drug for a purpose, and if the purpose is not to have a child, then for me it is the same as a diabetic who is taking insulin to process sugar. The pill is a drug, nothing else […].”* These perceptions were linked to external hormones in the OCP. Fannie noted, *“My period is back now, without hormones”,* indicating the absence of the OCP’s external hormones. Ida also shared, *“Hormonal contraception no longer suits me due to side effects and fluctuations. It no longer adds value to my life, so, it is no longer positive for me”.*

Some participants, such as Rachel, discontinued the pill after determining that it was unnecessary without the need for hormonal regulation*: “For me, it is not much different than before. Now I’m not giving my body hormones for no reason. Since the reason for taking hormones is gone, there is no logical reason to continue”.* Interviews highlighted the significant role of hormones in explaining physiological changes post-discontinuation. Lily, for example, stated, *“I took them again for a week, and my mood improved, which made me realize it was due to the loss of hormones”.* For Lily, stopping external hormones related to her previous experiences of discontinuation, confirming the impact of the OCP on the body. These changing values also indicate that the OCP is often associated with *“contraception and hormones”* (Carol).

*“The best thing I’ve ever done”* – Experiences

After stopping the pill, respondents overwhelmingly felt positive about their decision, noticing health improvements, such as the disappearance of side effects like migraines. Despite negative side effects post-discontinuation, for instance, Fannie remarked, *“In the end, I learned a lot and now I do not understand everything, but I understand a lot more and I […] Yes, I feel much better in general”*. Additionally, interviewees viewed discontinuation as an opportunity to understand their bodies better and feel more empowered. Lola explained, *“I feel good [laughing], I have to be honest. I feel like I know a little bit again: Who am I? And no longer: What could be a side effect of a hormone pill? Instead, I know that when I feel down, I truly am down”.* She added that while she feels empowered, her sexual freedom is reduced: *“It was a positive experience for me, and I feel more empowered. Even though I’m not as free now because I can no longer take the pill, I feel more natural and more in my skin”.*

For some, these positive feelings after discontinuation led to complete rejection of hormonal contraception: *“For me, it is the best thing I have ever done. I would never, ever, ever go back to the pill”* (Amira). However, others would consider taking the pill again if necessary, for instance, for *“practicability”* (Sophia) or health during menopause. Luca stated, *“[…] but truly only if there is no other option for my health. If I had to, if I had to suffer a lot, I would do it again”.* They researched information about discontinuation online and emphasised the need for more support and information about post-discontinuation issues (cf. biopsychological section): *“[…] as far as stopping the pill is concerned because there’s no real help for stopping the pill and […] yeah, even if you watch videos about what you could do, there’s nothing that truly, yeah, helps”* (Natalia). In addition to seeking more information, respondents expressed the need for safer and more multifaceted alternatives to contraceptive pills.

*“The mitigation”* – Relationships

The interviewees experienced both positive and negative aspects during the weaning process, receiving varying levels of support from their surroundings. They reported positive feedback about ceasing the OCP, particularly from their partners, who were generally kind and loving but struggled to understand mood swings and pain. Some partners were notably attentive and caregiving. Overall, the respondents felt fortunate and grateful to their supportive partners. Helena stated, *“He [her partner] was very kind and nice. He tried his best, of course, but I do not think he was able to assess where it was coming from and why I was feeling so bad at that time. And I could not truly put my finger on it”.* This narrative also reflects her partner’s insecurity regarding the symptoms after discontinuation. Some respondents, their mothers, and other family members also played important roles. Sophia reported discussing menstruation and hygiene with her mother and aunt: *“Then, I just wanted to talk about it, and I was able to do that with both of them. And that helped me a lot, especially talking to my mom again about how she was doing and picking up tips”.*

*“Successful at first because I did not get pregnant”* – The evaluation of their own contraceptive history

We inquired about the respondents’ contraceptive histories and their evaluations. They considered their experiences to be successful because of the absence of unintended pregnancies. Fannie described her journey as *“[…] Adventurous [laughing]. […] I mean it was okay, it was not dramatic, and I did not get pregnant or anything. It was definitely good”.* Although Fannie noted successful pregnancy prevention, her experiences were both positive and challenging.

Some respondents expressed regret over the introduction of the OCP: *“So, of all the decisions, it was the stupidest decision, just because I had no idea”* (Natalia). For others, the pill was *“the best alternative at the time”* (Amira), highlighting the young age of initiation and limited contraceptive options. Olive stated, *“I do not think the pill is the only solution*”. Other respondents echoed these sentiments, evaluating the pill’s significance both personally and for those at risk of pregnancy. However, they also highlighted the lack of adequate information throughout their experience with the OCP, from initiation to discontinuation.

## Discussion

This study revealed the multifaceted dynamics shaping decisions regarding OCP uptake, use, and discontinuation. Important themes, such as pregnancy prevention, side effects, and hormonal birth control, changed over the (dis)continuation experience. Incorporating a phase following discontinuation, which has rarely been explored in previous studies, provides a more detailed understanding of how users reflect on and reinterpret their experiences with contraceptives. This aligns with the contraceptive journey framework’s^6^ emphasis on temporality, yet it expands the concept by considering contraceptive use both cyclical and biographical. This approach highlights changing risk perceptions towards hormones, side effects, and pregnancy prevention. Analysed by a critical feminist methodology, these are deeply embedded in societal norms and expectations surrounding contraceptive and reproductive responsibilities. Therefore, this article adds to the understanding of informal knowledge and lived experiences (vernacular knowledge^43^ regarding the OCP. Additionally, although the first studies from the Netherlands^27^ and South Africa^7^ incorporated the CJ in their work, this is the first study to add the post-cessation phase. As past research^8^ has shown that factors influencing contraceptive choice consist of diverse global settings, our study adds an important analytical perspective to contraceptive research globally. This highlights the necessity for rights-based approaches that acknowledge individuals’ autonomy in contraceptive decision-making and regard personal needs as a human right.^45^ It also emphasises the importance of ensuring access to comprehensive information, non-coercive counselling, and a full range of contraceptive options, including the right to discontinue, switch, or choose a new method.^46^

Participants were directed towards the OCP through normalisation, which was influenced by their immediate social environment and gynaecologists, leading to the pill’s perception as the default contraceptive option. This phenomenon is consistent with the findings of other qualitative studies.^3,10,27^ Normalisation involves the feminisation of contraceptive responsibility, termed *“gendered compulsory birth control”* by Littlejohn.^3^ This perspective often attributes pregnancy prevention responsibility to women’s ability to give birth,^3,44^ limiting shared responsibilities between partners. Wigginton et al. argue that this extends beyond female-specific methods into broader gendered social processes.^4^ Male partners were absent from discussions on contraceptive choices during the initiation, although they became more involved in cessation decisions, suggesting increased partner responsibility with age and relationship progression. This pattern mirrors broader societal norms that not only influence contraceptive choices but also restrict the potential for shared responsibility between partners, underscoring the need for profound structural and relational changes. By fostering open dialogue and challenging gender roles, initiatives can promote equitable contraceptive decision-making. Healthcare providers play a crucial role in encouraging partner involvement and offering counselling regarding the relational aspects of contraception.

Gynaecologists influenced the OCP experience significantly, often prescribing OCPs as the default method without discussing alternatives or addressing concerns about side effects. Feminist scholars have criticised the biomedical power of medical professionals in contraceptive prescriptions,^23,33,34,47^ and our data corroborate their considerable medicalised authority over potential contraceptive users. This medicalised^48,49^ approach to contraception can potentially undermine sexual and reproductive autonomy and limit the ability to make fully informed decisions about contraceptive choices. As shown in previous research,^8^ user concerns, especially regarding side effects, are often dismissed, which can delay discontinuation or foster mistrust in healthcare providers. Our research, by focusing on these dynamics within a German setting, contributes to the expanding international literature advocating for a transition to rights-based, person-centred contraceptive care.^10,45,46^ This approach prioritises collaborative decision-making, acknowledges the value of personal knowledge, and offers counselling that aligns with the user's changing preferences and life circumstances.

Age played a crucial role in shaping participants’ perceptions of risk. Younger individuals focused on preventing pregnancy and were less worried about side effects, whereas older participants showed increased concerns about hormonal exposure and maintaining control over their bodies. This is corroborated by Carbone et al.,^54^ Geampana et al.,^19^ and Zettermark,^10^ which highlight how social positioning shapes contraceptive concerns. Zettermark’s findings, in particular, align with our data showing how older participants distance themselves from stigmatised groups such as *“irresponsible teenagers.”*^10^ This echoes common public health narratives that emphasise unplanned pregnancies and shape risk perceptions.^3^ Neoliberal societies promote the ideal of optimal pregnancy timing, facilitated by the OCP, enabling women to manage their career and reproductive goals.^4^ Consequently, the pill affects social reproduction significantly,^50,51^ influencing women’s identities and interactions with societal norms. Our study indicates that contraceptive responsibility and normalisation are closely linked to various aspects of the CJ.^6^

The participants in our study reported negative experiences due to the side effects of the OCP, initially switching to different OCPs. However, discomfort and attributing these side effects to the OCP often led to discontinuation, consistent with another Dutch study.^27^ The significance of side effects in the decision to discontinue the OCP has been demonstrated in qualitative,^20^ quantitative,^26,52,53^ and review^9,16^ studies. However, as Otte et al. (2023) noted, limited research exists on how and when users attribute these side effects to OCPs. Among our participants, this attribution occurred during or after OCP use, with certainty often forming post-discontinuation. This suggests that retrospective reflection plays a significant role in shaping users’ understanding of their contraceptive experiences. In this context, another study found that risk awareness evolves over time.^54^ Future studies should explore the mechanisms, such as temporal and experiential dimensions, underlying the attribution of side effects and risks to OCPs. Moreover, research can explore how social discourse, peer networks, and digital platforms influence users’ interpretations of bodily experiences when medical acknowledgment is lacking. Longitudinal approaches may provide insights into how individuals manage side effects, make decisions with providers, and adjust their contraceptive strategies.

An interesting shift in perceptions of risk, hormones, and safety was observed throughout the (dis)continuation process. Therefore, contraceptive safety is not a static category but rather a dynamic perception shaped by age, experience, and broader cultural narratives. We were able to construct an age effect in our data where participants did not initially consider side effects or the impact of external hormones, but after discontinuation and with older age, they emphasised the perceived risk of these side effects. The concept of contraceptive safety also evolved; initially, pregnancy prevention was paramount, but post-discontinuation, the focus shifted to the safety of the contraceptive itself regarding side effects. Despite limited research, Geampana’s analysis noted a shift towards nonhormonal options, which were perceived as safer and more manageable concerning side effects and health risks.^19^

In addition, the growing emphasis on natural and hormone-free contraceptive options in the context of OCPs discontinuation reflects a broader shift in attitudes towards hormonal contraception.^9,26^ Our analysis suggests that this trend is not merely a result of unfounded fears or misinformation but rather a complex interplay of personal experiences, health concerns, and a desire for more sustainable alternatives. Hormone-free options were not only seen as safer but also more aligned with participants’ personal values and evolving reproductive goals. The prevalence of hormone-related discussions on social media platforms underscores the significance of this issue in public discourse.^29,30,55^ While it may be tempting to dismiss this phenomenon as *“hormophobia”*,^56^ such a characterisation oversimplifies the underlying motivations and concerns of individuals seeking alternative contraceptive methods. Instead, our analysis adds to Thomé’s qualitative analysis of the rejection of hormonal contraception in France: the scarcity of viable, sustainable alternatives and lived experiences with side effects.^35^ Our biographical approach provides a more nuanced understanding of the complex decision-making process involved in contraceptive choices, highlighting the need for a more comprehensive and patient-centred approach to reproductive healthcare that addresses both the physical and emotional aspects of contraceptive use. Future research should explore the relationships among perceived risk and contraceptive safety perceptions. Additionally, investigating the development and accessibility of hormone-free contraceptive methods could inform innovation and policies for expanding contraceptive options and addressing unmet needs.

Our research also offers new perspectives on the process of OCP (dis)continuation, viewing it as both transitional and biographical. Discontinuing OCPs may also signify a developmental transition, such as deciding not to have children or choosing long-term contraceptives, in which potential pregnancy is no longer viewed negatively, particularly among older participants. Research on OCP (dis)continuation as a transitional process is lacking, and future studies should investigate the factors influencing transitional theories, including support and coping strategies. Further, the post-discontinuation phase, although often overlooked, is a crucial period in contraceptive journeys. It offers valuable insights into how users reflect on their experiences, reassess risks, and navigate through changing health priorities and reproductive goals. Future research should delve deeper into this phase to better understand long-term contraceptive decision-making, evolving attitudes towards hormonal methods, and the support structures users require when transitioning from contraception.

### Limitations

By utilising a qualitative methodology, we prioritise analytical depth over generalisability, with a small but sufficient sample size that enables rich, in-depth insights into diverse contraceptive experiences. Thematic saturation was achieved, supporting the adequacy of the number of participants. However, several limitations should be noted in our research. The participants included diverse socioeconomic backgrounds, ages, and geographic locations, but lacked racial diversity, with all participants being white and only one from another country. Prioritising social media recruitment, being a practical and strategic decision, likely excluded certain groups. Participants contacted us independently, so we cannot ascertain whether those who did not reach out had different experiences. Additionally, the project was unfunded, leading to financial and time constraints that may have affected recruitment and data saturation. Additional resources would have allowed us to expand recruitment into more diverse offline contexts, potentially capturing broader socioeconomic and regional diversity and enabling follow-up interviews or participatory approaches. Furthermore, while the contraceptive journey framework offers a useful structure for examining complex and evolving contraceptive experiences, it might have imposed some conceptual limitations, potentially restricting the emergence of themes beyond this framework. Also, with the overlap of factors in our conceptual framework, theme assignment was dependent on our research team’s coding.

The research team’s positionality, consisting mainly of white female German researchers with personal experience in using or stopping OCPs, influenced both the data collection and its interpretation. These shared experiences likely helped build rapport with participants and increased their sensitivity to subtle perspectives. However, they might also have influenced how interview questions were framed and how data were interpreted through specific experiential lenses.

## Conclusions

Our research indicates that reproductive responsibility, the normalisation of OCP use, the attribution of side effects to OCPs, evolving attitudes towards hormones and pregnancy prevention, and interactions with peers and gynaecologists influence the (dis)continuation process in Germany. It underscores the need to consider the informal knowledge and lived experiences with OCPs, including implementation, cessation, and post-cessation phases into future research, policy, and practice. Future studies should delve deeper into the mechanisms underlying the (dis)continuation of the pill.

## Supplementary Material

Supplementary Figure 1. Thematic map of the journey with the OCP in Germany.

## Footnotes

* All names are pseudonyms.

† We acknowledge that all individuals, regardless of their gender, should have the opportunity to access birth control. In this manuscript, we use the term “(former) pill users” when referring to individuals who took the OCP and when discussing it as a sexual and reproductive right. However, when referencing research, we used the term to describe the participants throughout the respective publication.
