# Supplementary material for: Between contraception and hormones: a qualitative analysis of the lived experiences of former contraceptive pill users
Source: Sex Reprod Health Matters. 2025 Sep 22;33(1):2563393. doi: 10.1080/26410397.2025.2563393 (PMC12548073; doi:10.1080/26410397.2025.2563393)
Supplement: Supplementary Figure 1. Thematic map of the journey with the OCP in Germany. [file ZRHM_A_2563393_SM5218.docx]

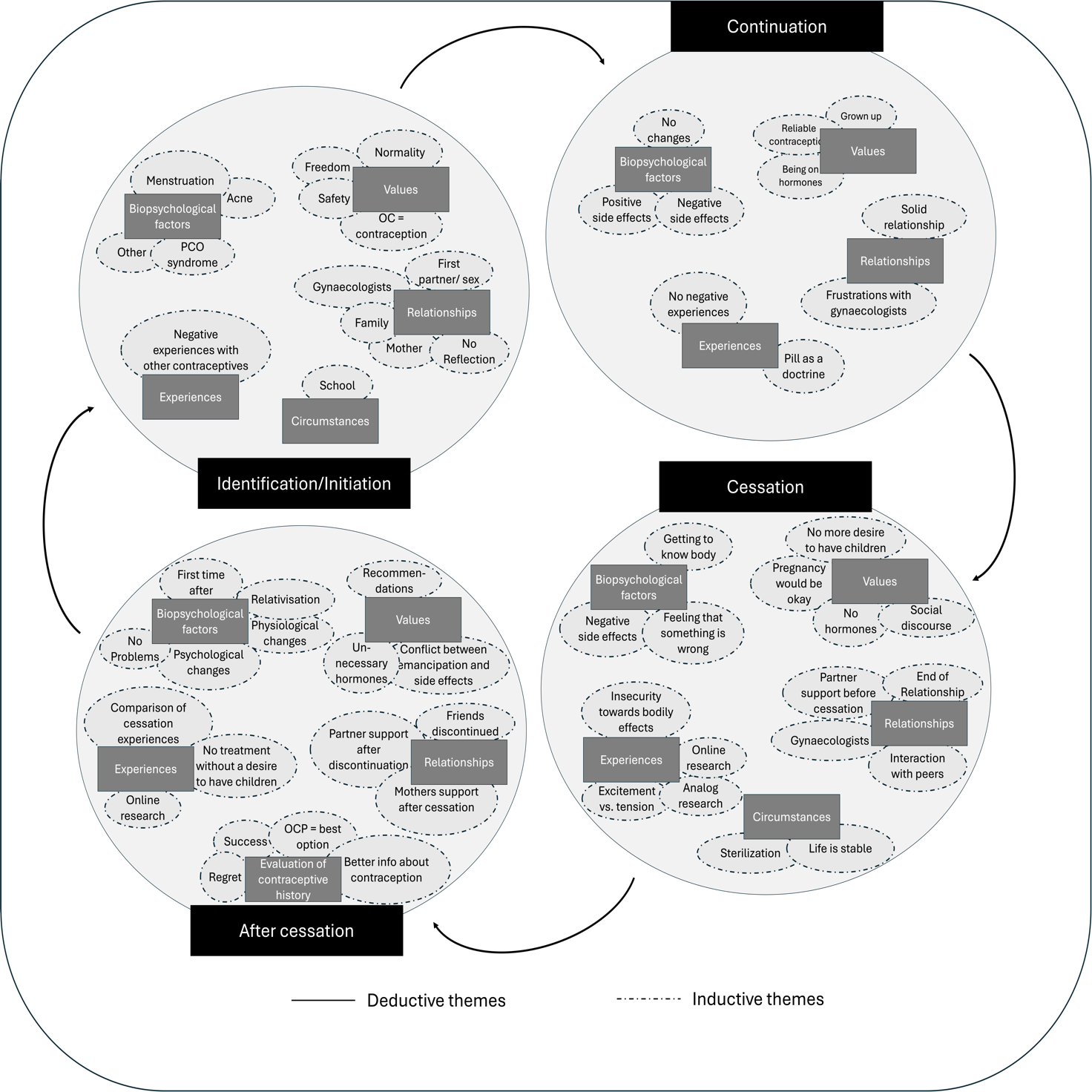


Supplementary Figure 1. Thematic map of the journey with the OCP in Germany (*Own illustration).*

Supplementary Figure 1 illustrates the themes of thematic analysis. The deductive themes are based on the theory of the contraceptive journey and interview guide (Simmons et al., 2023). Inductive themes were derived by JN and LG through an iterative coding and analysis process following Braun and Clarke (Braun & Clarke, 2019; Byrne, 2022).
